# Brilliant Record of a Newspaper Man

**Published:** 1904-06

**Authors:** 


### BRILLIANT RECORD OF A NEWSPAPER MAN.

The success which has attended William E. Curtis, the famous correspondent of *The Chicago Record–Herald,* is rarely attained by newspaper writers. Beginning his career in Chicago in 1872 as reporter, he rapidly rose to the position of managing editor. He resigned that position on receiving a government appointment as secretary of the South American commission. Mr. Curtis traveled extensively in Central and South America while in this position, producing several popular volumes as the result of his literary labors. Afterward co-operating with Secretary of State James G. Blaine, Mr. Curtis organized the work of the bureau of American republics, with the result that he was placed in charge of that organization, and at the World's Columbian Exposition he distinguished himself by his labors as the executive head of the Latin–American department. As correspondent of *The Chicago Record Herald,* Mr. Curtis' travels have carried him into every section of the United States as well as into all quarters of the globe. His China and Japan letters were published in book form; likewise his letters from England, Germany and France, as well as those written during his travels in Mexico and South America.

No newspaper correspondent possesses the facility shown by Mr. Curtis in writing on any of the diversified subjects embraced in his correspondence and making it luminous. Nor is any correspondent followed so closely year after year by the thousands of readers of *The Chicago Record-Herald.* On his recent trip to the Holy Land Mr. Curtis' letters have been read more closely than ever, and his descriptions of that interesting section of the globe as it appears today have been quoted everywhere.

A daily letter from Mr. Curtis appears in *The Chicago Record–Herald.*

